# Efficacy and Safety of Envafolimab Combined With Capecitabine and Lenvatinib as Postoperative Adjuvant Therapy in Resected Biliary Tract Cancer With High‐Risk Recurrence Factors: A Phase II Single‐Center Prospective Study

**DOI:** 10.1002/cam4.71756

**Published:** 2026-03-27

**Authors:** Yitong Tian, Tian Lei, Qingyang Ruan, Xueying Zhou, Ruijing Shen, Tianao Xie, Shijie Li, Xiujun Cai, Mingyu Chen

**Affiliations:** ^1^ Department of General Surgery, Sir Run‐Run Shaw Hospital Zhejiang University Hangzhou China; ^2^ National Engineering Research Center of Innovation and Application of Minimally Invasive Instruments Zhejiang University Hangzhou China; ^3^ The School of Medicine Zhejiang University Hangzhou China

**Keywords:** adjuvant triple treatment, biliary tract cancer, high‐risk recurrence factor, phase II trial

## Abstract

**Background:**

Biliary tract cancer (BTC) is an aggressive malignancy characterized by a high recurrence rate and poor postoperative prognosis, despite advances in adjuvant therapy. This phase II study evaluated the efficacy and safety of a novel adjuvant regimen combining envafolimab, lenvatinib, and capecitabine in patients with BTC at high risk of recurrence following R0 resection.

**Methods:**

This single‐center, open‐label, single‐arm phase II trial enrolled patients with high‐risk recurrence factors after curative resection. Patients received envafolimab (400 mg subcutaneously once every 3 weeks), Lenvatinib (8 mg orally once daily), and capecitabine (1000 mg/m^2^ orally twice daily, 2 weeks on/1 week off, continued without cycle limitation). The primary endpoint was disease‐free survival (DFS); secondary endpoints included overall survival (OS) and safety.

**Results:**

28 of 30 screened patients were included in the final analysis. The median DFS was 15.63 months, and the 1‐year DFS rate was 68.3%. Median OS was not reached but 1‐year OS was 91.4%. Treatment‐related adverse events (TRAEs) occurred in 17 patients, with grade 3/4 TRAEs observed in eight patients. No treatment‐related deaths were reported. Exploratory analysis suggested that baseline CA19‐9 levels were significantly associated with early recurrence.

**Conclusions:**

The adjuvant combination of envafolimab, lenvatinib, and capecitabine demonstrates promising efficacy and a manageable safety profile in high‐risk BTC patients after R0 resection. However, these findings still require validation in larger, multicenter, randomized controlled trials.

## Graphical abstract

A phase II single‐center prospective study evaluating envafolimab combined with capecitabine and lenvatinib as postoperative adjuvant therapy in resected biliary tract cancer with high‐risk recurrence factors. The regimen demonstrated encouraging efficacy (median DFS 15.6 months; 1‐year DFS 68.3%; 1‐year OS 91.4%) and manageable safety, with no treatment‐related deaths. Elevated CA19‐9 was associated with early recurrence.

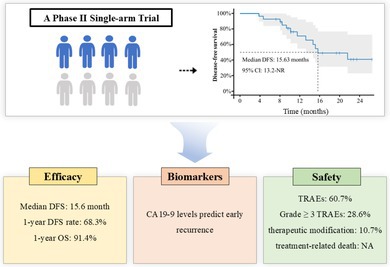

## 1 Introduction

Biliary tract cancers (BTCs) are a heterogeneous group of highly aggressive malignancies, encompassing distinct anatomical and biological subtypes, including intrahepatic cholangiocarcinoma (ICC), perihilar cholangiocarcinoma (pCCA), extrahepatic cholangiocarcinoma (ECC), and gallbladder cancer (GBC). These tumors are associated with a poor prognosis, particularly in patients with high‐risk features for recurrence [1, 2]. Despite recent advances in adjuvant therapy [1, 3, 4], current clinical guidelines uniformly recommend capecitabine as the standard first‐line adjuvant chemotherapy following resection [3, 4, 5, 6, 7]. However, survival outcomes remain suboptimal, highlighting a critical unmet need for more effective therapeutic strategies.

The integration of immunotherapy and targeted agents has transformed the treatment landscape for BTC, especially in advanced or unresectable disease [8, 9, 10]. Notably, these phase III trials (TOPAZ‐1 and KEYNOTE‐966) demonstrated that adding durvalumab or pembrolizumab to gemcitabine and cisplatin chemotherapy significantly improves overall survival, establishing ICIs as a new standard of care in the first‐line setting [11, 12]. Nevertheless, the magnitude of clinical benefit in these trials was modest, underscoring the need for further therapeutic optimization.

Envafolimab, a novel subcutaneously administered PD‐L1 inhibitor, represents the first approved drug of its kind in this route of administration and has been approved for the treatment of microsatellite instability‐high (MSI‐H) or mismatch repair‐deficient (dMMR) solid tumors [13, 14, 15, 16]. Retrospective analysis has shown clinically meaningful antitumor activity of envafolimab in unresectable solid tumors, including hepatocellular carcinoma and cholangiocarcinoma with a median progression‐free survival (PFS) of 5.4 months in the first‐line setting [17]. In a phase II trial, the combination of envafolimab and lenvatinib in unresectable hepatocellular carcinoma achieved a median overall survival of 18.5 months and a median PFS of 9.4 months, further supporting the potential of this dual approach [15].

Lenvatinib, a multi‐targeted tyrosine kinase inhibitor, inhibits vascular endothelial growth factor (VEGF) receptors 1–3, fibroblast growth factor (FGF) receptors 1–4, and other kinases, exerting potent antiangiogenic and immunomodulatory effects that may synergize with immune checkpoint blockade [18, 19]. Preclinical and clinical evidence suggests that combining anti‐angiogenic agents with immunotherapy can enhance antitumor immunity by normalizing the tumor microenvironment and improving T‐cell infiltration.

Building on these findings, we hypothesized that the combination of envafolimab, lenvatinib, and capecitabine could offer enhanced efficacy in the adjuvant setting for high‐risk BTC patients following R0 resection. In this phase II trial, we evaluated the clinical benefit of this triple‐combination regimen, with a primary focus on disease‐free survival. Our study aims to assess whether this innovative approach can improve postoperative outcomes and to explore potential biomarkers that may predict recurrence risk, thereby informing the development of personalized adjuvant strategies in BTC. We present this article in accordance with the TREND reporting checklist.

## 2 Method

### 2.1 Study Design

This was a single‐center, open‐label, single‐arm phase II trial designed to evaluate the efficacy and safety of adjuvant therapy with envafolimab, capecitabine, and lenvatinib in patients with high‐risk BTC after R0 resection.

### 2.2 Patient Eligibility

Eligible patients from Sir Run Run Shaw hospital were aged 18 years or older and met the following inclusion criteria: (1) histologically confirmed BTC; (2) complete surgical resection (R0) with negative margins; (3) presence of at least one high‐risk pathological feature for recurrence, such as lymph node metastasis, perineural invasion, or lymphovascular invasion; (4) no evidence of disease recurrence on postoperative imaging within 4 weeks of surgery; (5) no contraindications to immunotherapy; and (6) adequate organ function/biochemical parameters, defined as: Eastern Cooperative Oncology Group (ECOG) performance status ratings of 0 or 1; absolute neutrophil count ≥ 1.5 × 10^9^/L; hemoglobin concentration ≥ 90 g/L; platelet count ≥ 100 × 10^9^/L; total bilirubin < 1.5 times the upper limit of normal (ULN); aspartate transaminase (AST) and alanine transaminase (ALT) < 2.5 × ULN (or < 5 times ULN in patients with liver metastases); creatinine clearance > 60 mL/min (calculated using the Cockcroft–Gault formula); and left ventricular ejection fraction ≥ 50% on echocardiography.

Key exclusion criteria included: (1) active or clinically significant autoimmune disease requiring systemic immunosuppressive therapy, except for stable, asymptomatic conditions not requiring treatment, and (2) use of systemic or absorbable topical corticosteroids at doses exceeding 10 mg/day of prednisone or equivalent within 2 weeks before the first dose of study treatment for immunosuppressive purposes.

### 2.3 Treatment Protocol

Eligible patients received the triplet regimen starting within 4–8 weeks after surgery. The treatment consisted of Envafolimab (400 mg administered subcutaneously once every 3 weeks, for up to 35 cycles); Lenvatinib (8 mg orally once daily, for up to 8 cycles); Capecitabine (1000 mg/m^2^ orally twice daily for two weeks followed by one week rest, continued without a predefined cycle limit). Treatment continued until disease recurrence, development of unacceptable toxicity, patient withdrawal of consent, or completion of the protocol‐specified duration. Dose modifications/interruptions followed predefined criteria based on adverse event severity.

### 2.4 Efficacy Assessments

Tumor response was evaluated according to the Response Evaluation Criteria in Solid Tumors version 1.1 (RECIST v1.1) [20]. Baseline imaging included contrast‐enhanced CT or MRI of the chest/abdomen/pelvis. Subsequent assessments were performed every 12 weeks (±7 days) from the start of treatment. Imaging was reviewed for disease recurrence, and assessments continued until recurrence, death, discontinuation due to toxicity, or study withdrawal.

### 2.5 Safety Monitoring

Safety was assessed throughout the study via physical examination, laboratory tests, and electrocardiograms, conducted at each cycle. Treatment‐related adverse events (TRAEs) were graded using the National Cancer Institute Common Terminology Criteria for Adverse Events (version 5.0) [21]. Serious adverse events were recorded and reviewed by the study team.

### 2.6 Outcomes Measures

The primary endpoint was disease‐free survival, defined as the time from initiation of the triple therapy to disease recurrence or death from any cause, whichever occurred first. Patients without an event were censored at the date of the last follow‐up. Concomitantly, 6‐month and 1‐year disease‐free survival rates were also estimated. Secondary endpoints included overall survival, defined as the time from treatment initiation to death (censored at the last follow‐up if alive), 6‐month overall survival rate, and 1‐year overall survival rate. Safety profiles were evaluated in all treated patients.

### 2.7 Statistical Analysis

In this single‐center, exploratory phase II trial, the target sample size of 30 patients was determined a priori based on previous studies and clinical judgment, without a formal sample size calculation.

Continuous variables were presented as median and interquartile range. Categorical variables are reported as numbers and percentages. Survival outcomes were estimated using the Kaplan–Meier method. Prognostic factor analysis was conducted in two stages. First, univariable Cox proportional hazards models with log‐rank tests were used to screen candidate variables for association with DFS (significance threshold: *p* < 0.05). Variables that showed significance in univariable analysis, along with prespecified clinically relevant factors, were included in a multivariable Cox regression model to estimate adjusted hazard ratios (HRs) and 95% CIs.

In exploratory analysis, the association between baseline tumor marker levels (CA19‐9, CA125, CEA) and early recurrence (ER, defined as disease recurrence within one year) was evaluated using Fisher's exact test. Predictive performance was assessed via receiver operating characteristic (ROC) curve analysis, and the area under the curve (AUC) was calculated. All statistical tests were two‐sided, with a significance level set at *p* < 0.05. Analyses were performed using R software (version 4.3.0) using *survival*, *survminer*, and *boot* packages and SPSS software (version 30.0).

## 3 Results

### 3.1 Baseline Characteristics

**FIGURE 1:**
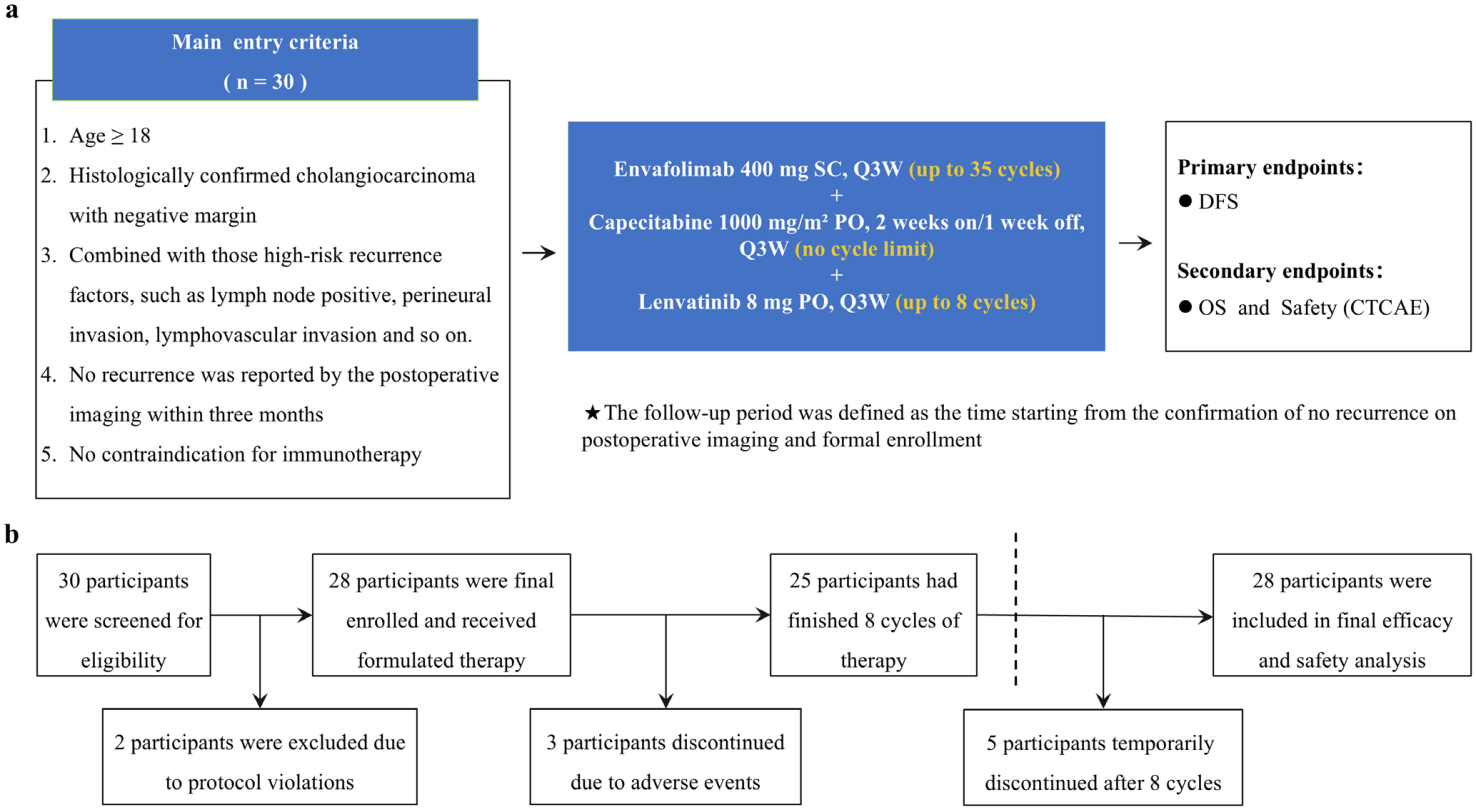
Study design and flowchart. (a) Eligibility criteria, treatment regimen, and endpoints. (b) Study flowchart. CTCAE, common terminology criteria for adverse events; DFS, disease‐free survival; OS, overall survival; PO, peros; Q3W, every 3 weeks; SC, subcutaneous.

A total of 30 patients with R0‐resected BTC were enrolled, with a median follow‐up duration of 18.38 months. Two patients discontinued treatment due to protocol deviations and were excluded from the final analysis. Specifically, the two patients showed an increase in tumor marker levels within three months after enrollment and subsequently requested a switch to intravenous regimen (AG regimen); therefore, they were excluded according to the study eligibility criteria. As a result, 28 patients were selected in the final analysis, including three patients who discontinued due to adverse events. The median duration of lenvatinib treatment was 5.3 months. Five patients discontinued capecitabine after 8 cycles with a median of 9.5 months, while the remaining patients continued therapy. All patients are still on envafolimab, with no discontinuations reported (Figure 1). The cohort comprised 17 men and 11 women, with a median age of 64 years. Tumor subtypes included ICC (25.0%), pCCA (10.7%), ECC (25.0%), and GBC (39.3%). All patients had at least one high‐risk pathological feature for recurrence: lymph node metastasis in 64.3%, perineural invasion in 60.7%, and lymphovascular invasion in 57.1%. The comprehensive baseline characteristics encompassing perioperative demographic parameters and histopathological profiles are systematically summarized in Table 1.

**TABLE 1:** Baseline characteristics of 28 patients.

	Capecitabine + lenvatinib + envafolimab (*n* = 28)
Age (years), median (range)	64 (47–83)
≤ 65	17 (60.7)
> 65	11 (39.3)
Gender, *n* (%)	
Male	17 (60.7)
Female	11 (39.3)
ECOG Performance status, *n* (%)	
0	13 (46.4)
1	15 (53.6)
Tumor Size (cm), median (range)	2.2 (0.4–8.5)
Tumor Location, *n* (%)	
Intrahepatic bile duct	7 (25.0)
Extrahepatic bile duct	7 (25.0)
Perihilar	3 (10.7)
Gallbladder	11 (39.3)
Differentiation, *n* (%)	
Poor	12 (42.9)
Moderate	14 (50.0)
Well	2 (7.1)
Histology type, *n* (%)	
Adenocarcinoma	27 (96.4)
Adenosquamous carcinoma	1 (3.6)
Tumor stage, *n* (%)	
T1	12 (42.9)
T2	10 (35.7)
T3	6 (21.4)
Lymph node stage, *n* (%)	
N0	12 (42.9)
N1	14 (50.0)
N2	2 (7.1)
Cholangioenterostomy, *n* (%)	12 (42.9)
Tumor‐specific markers (CA199/CA125/CEA), *n* (%)	
Both Normal^#^	7 (25.0)
After Normal	11 (39.3)
Before Normal	1 (3.6)
Neither Normal	9 (32.1)
Lymph node, *n* (%)	
Positive	18 (64.3)
Negative	10 (35.7)
Neural Invasion, *n* (%)	
Positive	18 (64.3)
Negative	10 (35.7)
Lymphovascular tumor embolus, *n* (%)	
Positive	16 (57.1)
Negative	12 (42.9)
Number of high‐risk recurrence factors, *n* (%)	
1	12 (42.9)
2	7 (25.0)
3	9 (32.1)

*Note:* Data are median (range) or *n* (%). ECOG, Eastern Cooperative Oncology Group. CA 19–9, carbohydrate antigen 19–9.

### 3.2 Efficacy Outcomes

**FIGURE 2:**
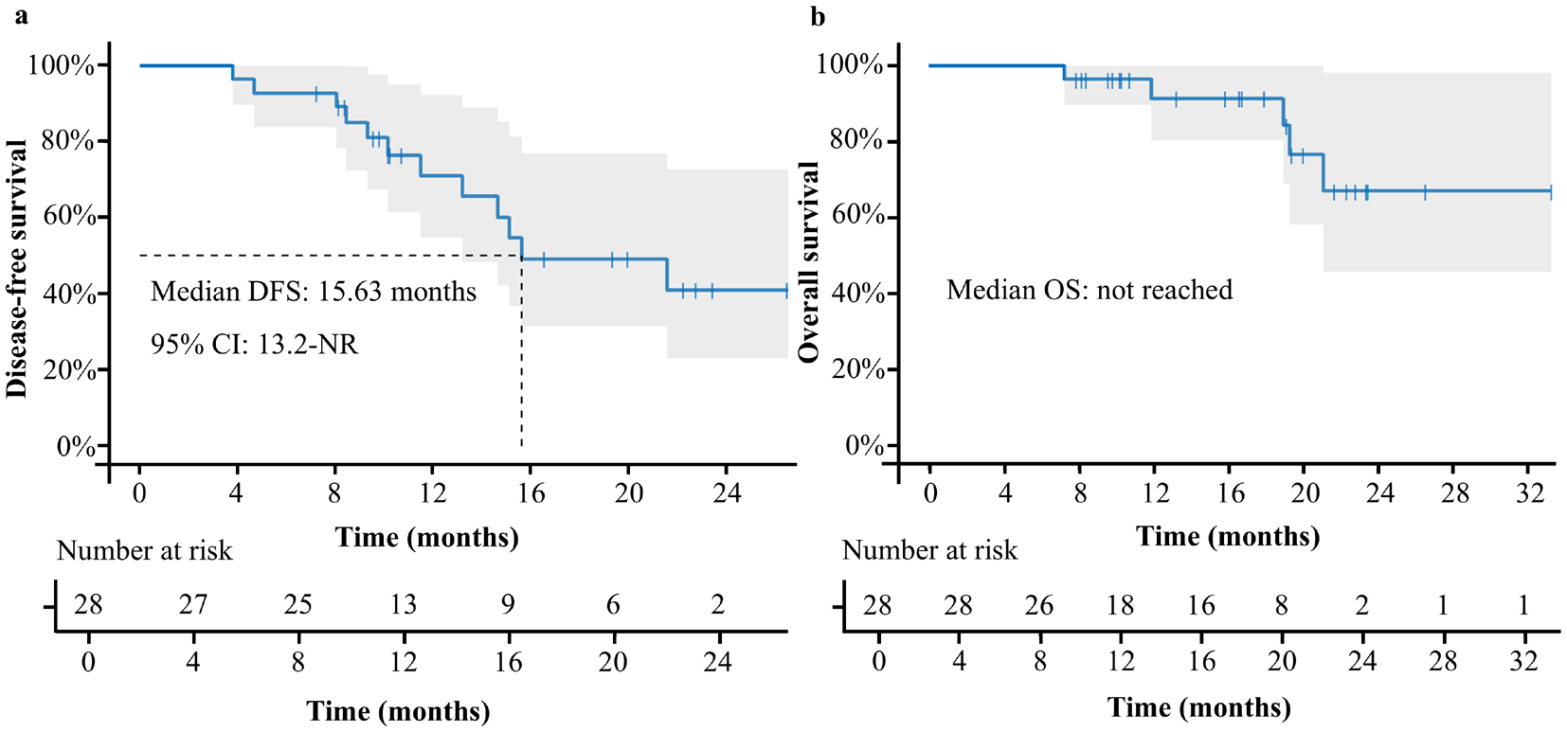
DFS and OS in the final analysis. (a) Kaplan–Meier estimates disease‐free survival of 28 eligible patients. (b) Kaplan–Meier estimates overall survival of 28 eligible patients. CI, confidence interval.

**FIGURE 3:**
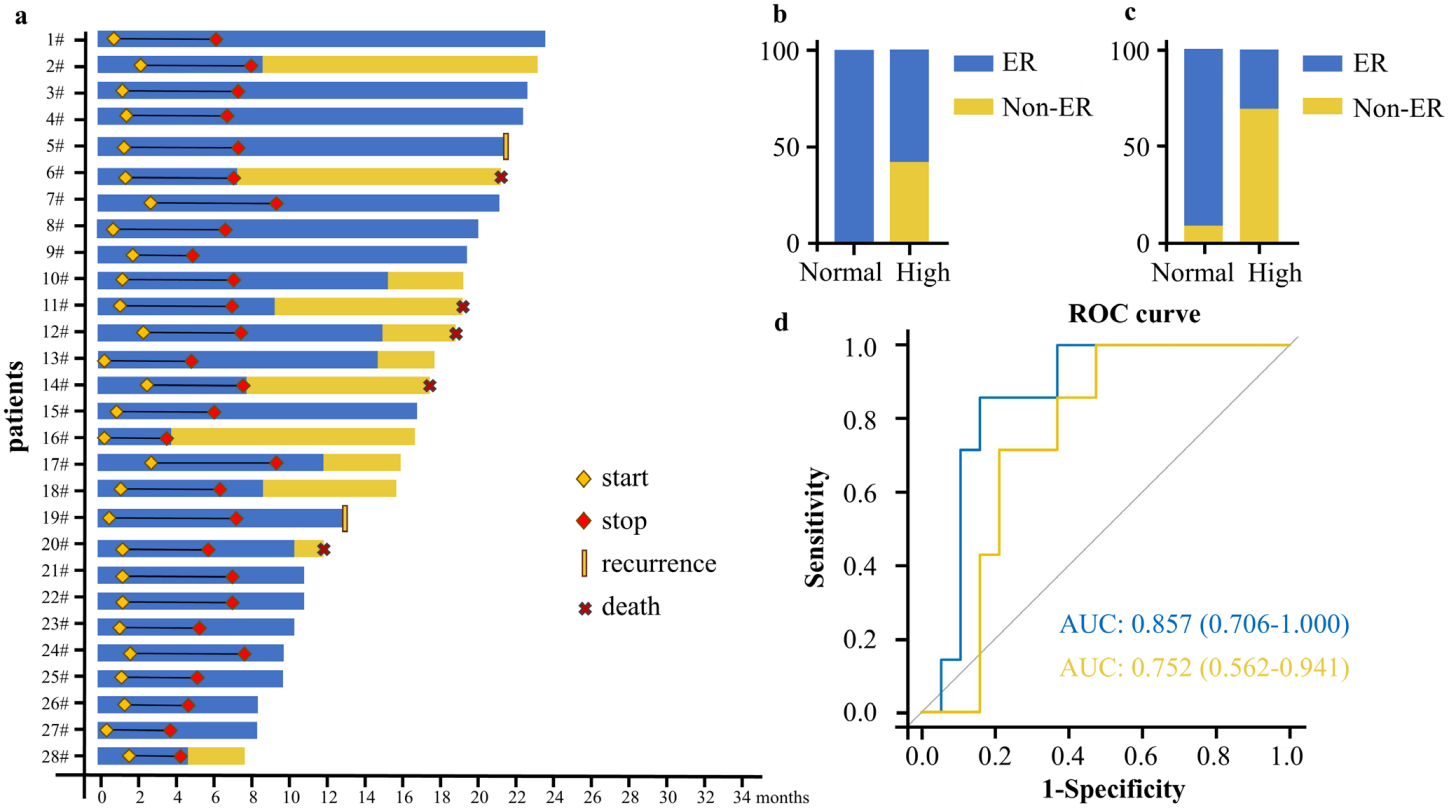
Disease course, treatment timeline, and subgroup biomarker analysis. (a) Disease course and treatment timelines for each patient. (b) Distribution proportions of ER and Non‐ER in the normal and high CA19‐9 groups before operation. (c) Distribution proportions of ER and Non‐ER in the normal and high CA19‐9 groups after the first triple therapy. (d) Comparison of ROC curves for preoperative (yellow) and post‐first triple therapy (blue) CA19‐9 levels in predicting early recurrence. AUC, area under the ROC curve; CA 19–9, carbohydrate antigen 19–9. ER, early recurrence. Non‐ER, non‐early recurrence. ROC, receiver operating characteristic.

Among the 28 patients receiving the triplet regimen, 13 patients experienced disease recurrence at the study endpoint. The median DFS was 15.63 months. Kaplan–Meier estimates showed a 6‐month DFS rate of 92.9% and a 1‐year DFS rate of 68.3% (Figure 2a). A stepwise reduction in DFS was observed with an increasing number of high‐risk factors. For patients with only one risk factor, median DFS was not reached but exceeded the median DFS of the overall cohort (15.63 months). In contrast, DFS shortened with increasing numbers of risk factors, with a median DFS of 14.67 months in patients with two factors and 11.50 months in those with three factors. As of the data cutoff, five patients had died. The median overall survival (OS) has not been reached. Kaplan–Meier estimates showed a 6‐month OS rate of 100% and a 1‐year OS rate of 91.4% (Figure 2b). Figure 3a illustrates individual patient trajectories, including time from surgery to recurrence, treatment initiation and discontinuation, and survival status.

### 3.3 Prognostic Factor Analysis

Univariable Cox regression analyses identified four variables significantly associated with shorter DFS (*p* < 0.05): tumor size (*p* = 0.001), preoperative tumor marker levels (CA19‐9, CA125, CEA; *p* = 0.0004), lymph node metastasis (*p* = 0.015), and number of high‐risk recurrence factors (*p* = 0.022) (Table 2). The other two factors, perineural invasion and lymphovascular invasion, did not reach statistical significance.

**TABLE 2:** Prognostic factor analysis.

Variables	*N*	Univariable analysis	Multivariable analysis	
		*p*	HR (95% CI)	*p*
Age (years)				
≤ 65	17 (60.7)	0.080		
> 65	11 (39.3)			
Gender, *n* (%)				
Male	17 (60.7)	0.815		
Female	11 (39.3)			
ECOG Performance status, *n* (%)				
0	13 (46.4)	0.443		
1	15 (53.6)			
Tumor Size (cm), median (range)	2.2 (0.4–8.5)	0.001	0.84 (0.59–1.20)	0.336
Tumor location, *n* (%)				
Intrahepatic bile duct	7 (25.0)	0.586		
Extrahepatic bile duct	7 (25.0)			
Perihilar	3 (10.7)			
Gallbladder	11 (39.3)			
Differentiation, *n* (%)				
Poor	12 (42.9)	0.359		
Moderate	14 (50.0)			
Well	2 (7.1)			
Tumor stage, *n* (%)				
T1	12 (42.9)	0.253		
T2	10 (35.7)			
T3	6 (21.4)			
Lymph node stage, *n* (%)				
N0	12 (42.9)	0.070		
N1	14 (50.0)			
N2	2 (7.1)			
Tumor‐specific markers (CA199/CA125/CEA), *n* (%)				
Both Normal^#^	7 (25.0)	0.0004	2.63 (1.11–6.24)	0.028
After Normal	11 (39.3)			
Before Normal	1 (3.6)			
Neither Normal	9 (32.1)			
Cholangioenterostomy	12 (42.9)	0.937		
Lymph node, *n* (%)				
Positive	18 (64.3)	0.015	1.50 (0.22–10.17)	0.678
Negative	10 (35.7)			
Neural Invasion, *n* (%)				
Positive	18 (64.3)	0.958		
Negative	10 (35.7)			
Lymphovascular tumor embolus, *n* (%)				
Positive	16 (57.1)	0.231		
Negative	12 (42.9)			
Number of high‐risk recurrence factors, *n* (%)				
1	12 (42.9)	0.022	1.73 (0.75–4.00)	0.200
2	7 (25.0)			
3	9 (32.1)			

*Note:* The univariable log‐rank test was firstly implemented with a *P* < 0.05 significance threshold. Multivariate analysis used the Cox proportional hazard regression model.

Abbreviations: CA 19–9, carbohydrate antigen 19–9; CI, confidence interval; ECOG, Eastern Cooperative Oncology Group; HR, hazard ratio.

In multivariable analysis adjusting for these significant univariable predictors, elevated tumor marker levels remained an independent prognostic factor for worse DFS (*p* = 0.028; HR: 2.63; 95% CI: 1.11–6.24) (Table 2).

### 3.4 Biomarker Analysis

Given the significance of tumor markers in the prognostic model, we sought to further elucidate their relationship with early recurrence (ER) with an exploratory analysis. Preoperative CA19‐9 levels were strongly associated with ER (Table S1): no patient in the normal CA19‐9 group (< 37 U/mL) experienced early recurrence, whereas 57.9% of those with elevated levels recurred within 12 months (Fisher's exact test, *p* = 0.029; Figure 3b). After the first cycle of combination therapy, CA19‐9 levels further stratified risk: only 10.5% of patients with normalized or low post‐treatment levels had ER, compared to 71.4% in the elevated group (*p* = 0.006; Figure 3c).

Receiver operating characteristic (ROC) curve analysis demonstrated that CA19‐9 levels after the first treatment cycle had superior predictive performance for early recurrence (AUC = 0.857; *p* = 0.0033), with an optimal cutoff of 27.2 U/mL (sensitivity: 85.7%, specificity: 84.2%). Preoperative CA19‐9 levels also predicted ER but with lower discriminatory power (AUC = 0.752; *p* = 0.03), optimal cutoff: 39.3 U/mL (sensitivity: 100%, specificity: 52.6%) (Figure 3d).

### 3.5 Safety

All patients were evaluable for safety. Treatment‐related adverse events (TRAEs) occurred in 17 patients, including grade ≥ 3 TRAEs in eight patients. (Table 3). Notably, three patients required dose modifications or treatment discontinuation within 8 cycles: two discontinued lenvatinib due to severe palmar‐plantar erythrodysesthesia syndrome, according to the pre‐specified study protocol, and one discontinued both lenvatinib and capecitabine due to treatment‐related fever, fatigue, and cholangitis. After discontinuation of the respective medications, the adverse events in all three patients improved. No treatment‐related deaths occurred.

**TABLE 3:** Summary of treatment‐related adverse events.

Events	No, %
Any TRAEs	17 (60.7)
Any Grade ≥ 3 TRAEs	8 (28.6)
Treatment discontinuation due to TRAEs	3 (10.7)
Deaths due to TRAEs	0 (0.0)
No. of events of each TRAE	
Fever	7 (25.0)
Diarrhea	5 (17.9)
Disease progression	5 (17.9)
Anemia	5 (17.9)
Thrombocytopenia	5 (17.9)
Leukopenia	4 (14.3)
Palmar‐plantar erythrodysesthesia syndrome	4 (14.3)
Vomiting	3 (10.7)
Fatigue	3 (10.7)
Cecal hemorrhage	2 (7.1)
Mucositis oral	2 (7.1)
Chills	2 (7.1)
Belching	1 (3.6)
Bloating	1 (3.6)
Dry mouth	1 (3.6)
Dyspepsia	1 (3.6)
Gastroesophageal reflux disease	1 (3.6)
Upper gastrointestinal hemorrhage	1 (3.6)
Cholangitis	1 (3.6)
Autoimmune disorder	1 (3.6)
Neutropenia	1 (3.6)
Myalgia	1 (3.6)
Dizziness	1 (3.6)
Paresthesia	1 (3.6)
Pharyngitis	1 (3.6)
Rash maculo‐papular	1 (3.6)

*Note:* Data are reported as no (%). Reported TRAEs were limited to those occurring during treatment or ≤ 30 days post‐treatment. For recurrent TRAEs within a category, patients were counted once per category. Patients experiencing multiple distinct TRAEs contributed once to overall incidence totals.

Abbreviation: TRAEs, Treatment‐Related Adverse events.

## 4 Discussion

To our knowledge, this is the first clinical trial to evaluate the integration of an (ICI), a multi‐kinase inhibitor, and chemotherapy in the postoperative management of BTC. In this single‐arm phase II study, the triplet regimen—envafolimab, lenvatinib, and capecitabine—demonstrated clinically meaningful efficacy. Importantly, the regimen was associated with a manageable safety profile, with no treatment‐related deaths observed.

In the landmark BILCAP trial, adjuvant capecitabine demonstrated a survival benefit over observation, achieving a median overall survival of 51.1 months and a median recurrence‐free survival of 36.4 months in the intention‐to‐treat analysis [22]. However, the magnitude of benefit was modest, and recurrence rates remained substantial, underscoring the need for more effective strategies, particularly in high‐risk populations [23]. In contrast, our study reported a median DFS of 15.63 months and a 1‐year DFS rate of 68.3% in a cohort enriched for high‐risk features (lymph node metastasis, perineural invasion, or lymphovascular invasion), with shorter follow‐up and different efficacy endpoints. Despite these differences, our findings suggest an early disease control signal in this high‐risk population. Overall survival data remain immature, with the median OS not yet reached and a 1‐year OS rate of 91.4%. Recent studies, including the ASCOT trial, suggest alternative adjuvant chemotherapy regimens as potential options for BTC [24]. Notably, in the ACCORD trial, adding PD‐1 inhibitor camrelizumab to chemoradiotherapy significantly reduced the risks of death and recurrence in patients with resected high‐risk ECC or GBC [25], but not including perineural invasion, which was associated with poor outcomes across different BTC subtypes [26, 27, 28]. In contrast, the STAMP trial showed no survival benefit from chemotherapy intensification with gemcitabine plus cisplatin compared with capecitabine in node‐positive ECC, suggesting the limited efficacy of chemotherapy‐based escalation in this setting [29]. In this context, our findings provide hypothesis‐generating evidence that a triplet approach may offer improved early disease control for patients at high risk of recurrence.

Preclinical evidence further supports the rationale for combining ICIs with anti‐angiogenic agents and chemotherapy. VEGF overexpression contributes to immune evasion in BTC, and VEGF inhibition may synergize with immune checkpoint blockade [30]. Benmebarek [31] et al. demonstrated that VEGFR inhibition enhances the immunomodulatory effects of PD‐L1 blockade in BTC models, while Peng [32] et al. found that dual inhibition of FGFR and VEGFR pathways modulates PD‐L1 expression in ICC, suggesting a mechanistic basis for synergy. Nevertheless, not all clinical data uniformly support the benefit of adding ICIs. Two retrospective studies reported no significant survival advantage with the inclusion of ICIs in the adjuvant setting [33, 34]. Given the limited number of completed prospective trials, the role of combination immunotherapy in adjuvant BTC remains investigational.

In our study, we only selected patients who underwent R0 resection with high recurrence risks and excluded R1 patients because of potential tolerance issues from the triplet regimen. To mitigate potential toxicity from the triplet regimen, we devised the dosing regimen to ensure safety and efficacy based on the study objectives. Firstly, the dosage of Envafolimab was recommended in the first‐in‐human phase I study at 300 mg Q3W or 400 mg Q4W [35]. The Q3W schedule aligns with chemotherapy cycles, and previous studies have used 400 mg, supporting the dosage in this study [36]. Secondly, the capecitabine dose was reduced from the guideline‐recommended 1250 mg/m^2^ to 1000 mg/m^2^ (80% of the standard dose) without limit because the potential benefits of long‐term low‐dose therapy, known as metronomic chemotherapy [37], have shown promising efficacy in various malignancies, including breast, nasopharyngeal, and colorectal cancers [38, 39, 40].

The safety profile was consistent with the known toxicities of the individual agents [22, 41, 42]. Over 60% of patients experienced treatment‐related adverse events (TRAEs), including grade ≥ 3 TRAEs in 8 patients. Thrombocytopenia was the most frequent severe toxicity and was consistent with chemotherapy‐induced myelosuppression [22]. Notably, the incidence of grade 3–4 TRAEs was 44% in the BILCAP trial [22], which was higher than the 28% observed in the present study. Several factors may explain this difference: First, all patients in our study underwent R0 resection and had an ECOG performance status of 0–1, whereas the BILCAP trial included patients with other resection statuses. Second, the dose of capecitabine was reduced in our protocol (from guideline‐recommended 1250 mg/m^2^ to 1000 mg/m^2^). In addition, the small sample size and limited follow‐up may have restricted the detection of infrequent or late‐onset adverse events.

In our prognostic modeling, tumor‐specific markers emerged as the only independent predictor of DFS (*p* = 0.041). In subsequent exploratory subgroup analyses, we assessed these markers at three timepoints: preoperatively, pre‐treatment, and after the first cycle of therapy. We found that both preoperative and post‐treatment CA19‐9 levels were strongly associated with early recurrence. CA19‐9 has been widely included in multiple models for predicting disease recurrence [43]. Previous studies incorporated CA19‐9 into a multivariable CART model and reported an AUC of 0.728 [44]. Similarly, nomogram models combining CA19‐9 with tumor‐related variables achieved a C‐index of 0.777 in the training cohort and 0.716 in the validation cohort [45]. More recently, an interpretable XGBoost machine‐learning approach showed AUCs of 0.76 in the training cohort and 0.72 in the validation cohort [46]. These findings further support the contribution of CA19‐9 within more complex predictive frameworks. In our study, preoperative and post‐treatment CA19‐9 yielded AUCs of 0.752 and 0.857, comparable to prior reports. These findings support the potential prognostic value of CA19‐9 in high‐risk populations, with early changes reflecting treatment response.

Elevated CA19‐9 has been linked to molecular features associated with aggressive biology [47, 48], including *KRAS* mutations, enhanced glycolysis, epithelial–mesenchymal transition (EMT), and angiogenesis [49], which may explain its prognostic value. These findings support the integration of serial tumor marker monitoring into clinical decision‐making.

Several limitations must be acknowledged. First, the single‐center design may introduce selection bias. Second, the single‐arm structure precludes direct comparison with standard‐of‐care or control groups, limiting causal inference. Third, the small sample size reduces statistical power, particularly for multivariable modeling, where the event‐to‐variable ratio fell below the recommended 10:1 threshold, increasing the risk of type II errors. Additionally, clinical homogeneity among high‐risk patients may have limited the ability to detect subtle differences between subgroups.

Despite these limitations, this study represents the first phase II evaluation of a triplet adjuvant regimen combining an ICI (envafolimab), a multi‐kinase inhibitor (lenvatinib), and chemotherapy in patients with resected high‐risk BTC. The regimen demonstrated encouraging disease‐free survival outcomes with a manageable safety profile. Although overall survival data remain immature, these findings support further investigation of this multimodal strategy in larger, multicenter randomized trials with longer follow‐up. Biomarker‐driven strategies, incorporating dynamic CA19‐9 changes and emerging functional imaging modalities such as PD‐1/PD‐L1 PET probes [50], may further refine postoperative risk stratification and improve patient selection, thereby enhancing therapeutic precision and potentially improving clinical outcomes.

## Author Contributions

**Qingyang Ruan:** writing – original draft, writing – review and editing, validation, visualization, investigation, software, data curation, formal analysis. **Tianao Xie:** writing – original draft, writing – review and editing, validation, investigation. **Yitong Tian:** writing – review and editing, writing – original draft, investigation, visualization, validation, software, data curation, formal analysis. **Mingyu Chen:** writing – original draft, writing – review and editing, conceptualization, methodology, supervision, project administration, funding acquisition, resources. **Shijie Li:** writing – original draft, writing – review and editing, conceptualization, methodology, supervision, project administration, funding acquisition, resources. **Ruijing Shen:** writing – review and editing, writing – original draft, validation, investigation. **Tian Lei:** writing – original draft, writing – review and editing, software, formal analysis, data curation, validation, visualization, investigation. **Xueying Zhou:** writing – original draft, writing – review and editing, validation, investigation.

## Funding

This work was supported by the Natural Science Foundation of Zhejiang Province (Grant No. LR25H160001) and the Fundamental Research Funds for the Central Universities (Grant No. 226–2025‐00172).

## Ethics Statement

The authors are accountable for all aspects of the work in ensuring that questions related to the accuracy or integrity of any part of the work are appropriately investigated and resolved. The study was conducted in accordance with the Declaration of Helsinki and its subsequent amendments. The protocol was approved by the Ethics Committee of Sir Run Run Shaw Hospital (Approval No. 20230397). The patient's guardians independently selected the procedure and provided written consents.

## Conflicts of Interest

All authors have completed the ICMJE uniform disclosure form. The authors have no conflicts of interest to declare. This study was supported by a research grant from Simcere Zaiming Pharmaceutical Company Limited. The sponsor had no role in the design and conduct of the study; collection, management, analysis, or interpretation of data; preparation, review, or approval of the manuscript; or the decision to submit the manuscript for publication.

## Supporting information


**Data S1:** The summary of the study protocol.

**Table S1:** Fisher's exact tests for the relationship between tumor marker status at different time points (before operation/triple therapy initiation/post‐first triple therapy) and ER risk.

## Data Availability

De‐identified individual participant data that underlie the results reported in this article will be available upon reasonable request to the corresponding author after publication, following approval by the institutional ethics committee and the signing of a data use agreement. Statistical analysis code will be made available upon reasonable request.
