# Supplementary material for: Efficacy and Safety of Envafolimab Combined With Capecitabine and Lenvatinib as Postoperative Adjuvant Therapy in Resected Biliary Tract Cancer With High‐Risk Recurrence Factors: A Phase II Single‐Center Prospective Study
Source: Cancer Med. 2026 Mar 27;15(4):e71756. doi: 10.1002/cam4.71756 (PMC13140624; doi:10.1002/cam4.71756)
Supplement: Supplementary file 1 — Data S1: The summary of the study protocol. [file CAM4-15-e71756-s001.docx]

| Primary sponsor： | Sir Run-Run Shaw Hospital, Zhejiang University | | | | | | |
| --- | --- | --- | --- | --- | --- | --- | --- |
| Primary sponsor's address： | 3 Qingchun Road East, Hangzhou, Zhejiang, China | | | | | | |
| Secondary sponsor： | Country： | China | Province： | Zhejiang | City： | | Hangzhou |
|  | Institution hospital： | Sir Run-Run Shaw Hospital, Zhejiang University | | Address： | 3 Qingchun Road East, Hangzhou, Zhejiang, China | | |
| Source(s) of funding： | Sir Run-Run Shaw Hospital, Zhejiang University | | | | | | |
| Target disease： | Biliary tract cancers | | | | | | |
| Study type： | Interventional study | | | | | | |
| Study phase： | 2 | | | | | | |
| Study design： | Single arm | | | | | | |
| Objectives of Study： | To assess the safety and efficacy of envolizumab combined with lenvatinib and capecitabine for biliary tract cancers with high-risk recurrence after surgery. | | | | | | |
| Inclusion criteria: | 1) histologically confirmed BTC; 2) complete surgical resection (R0) with negative margins; 3) presence of at least one high-risk pathological feature for recurrence, such as lymph node metastasis, perineural invasion, or lymphovascular invasion; 4) no evidence of disease recurrence on postoperative imaging within 4 weeks of surgery; 5) no contraindications to immunotherapy; and 6) adequate organ function/biochemical parameters, defined as: Eastern Cooperative Oncology Group (ECOG) performance status ratings of 0 or 1; absolute neutrophil count ≥1.5×10⁹/L; hemoglobin concentration ≥90 g/L; platelet count ≥100×10⁹/L; total bilirubin <1.5 times the upper limit of normal (ULN); aspartate transaminase (AST) and alanine transaminase (ALT) <2.5×ULN (or <5 times ULN in patients with liver metastases); creatinine clearance >60 mL/min (calculated using the Cockcroft-Gault formula); and left ventricular ejection fraction ≥50% on echocardiography. | | | | | | |
| Exclusion criteria： | 1) active or clinically significant autoimmune disease requiring systemic immunosuppressive therapy, except for stable, asymptomatic conditions not requiring treatment; and 2) use of systemic or absorbable topical corticosteroids at doses exceeding 10 mg/day of prednisone or equivalent within 2 weeks before the first dose of study treatment for immunosuppressive purposes. | | | | | | |
| Interventions: | Group： | Envolizumab combined with lenvatinib and capecitabine group | | Sample size： | | 30 | |
|  | Intervention： | 1. Envolimab: 400mg, subcutaneous injection, Q3W, for 1 cycle of treatment, a total of 35 cycles. 2. lenvatinib: 8 mg orally, qd, a total of 8 cycles. 3. Capecitabine: 1000mg/m^2^ orally, bid, 2 weeks followed by discontinuation of the drug for 1 week, for 1 cycle of treatment, no cycle limit. | | | | | |
| Countries of recruitment and research settings： | Country： | China | Province： | Zhejiang | City： | | Hangzhou |
|  | Institution hospital： | Sir Run-Run Shaw Hospital, Zhejiang University | | Level of the institution： | Tertiary A | | |
| Outcomes： | Outcome： | Disease-free survival | | Type： | Primary indicator | | |
|  | Outcome： | Overall survival | | Type： | Secondary indicator | | |
|  | Outcome： | Adverse events | | Type： | Secondary indicator | | |
|  | Outcome： | Severe adverse events | | Type： | Secondary indicator | | |
| Collecting sample(s) from participants： | Sample Name： | | | Not appliable | | | |
| Participant age： | Min age:18 years | | | | | | |
|  | Max age: no limit | | | | | | |
| Gender： | Both | | | | | | |
| Randomization Procedure (please state who generates the random number sequence and by what method)： | After patients who met the inclusion and exclusion criteria signed the informed consent, they were given corresponding numbers according to the order of the visits, and treated with envolizumab combined with lenvatinib and capecitabine. | | | | | | |
| Calculated Results after the Study Completed public access: | Public | | | | | | |
| IPD sharing | Yes | | | | | | |
| The way of sharing IPD”(include metadata and protocol, If use web-based public database, please provide the url)： | All original data are available upon reasonable request to the corresponding authors. | | | | | | |
| Data collection and Management (A standard data collection and management system include a CRF and an electronic data capture： | A standard data collection and management system of an electronic data capture. | | | | | | |
| Data and Safety Monitoring Committee： | Not yet | | | | | | |


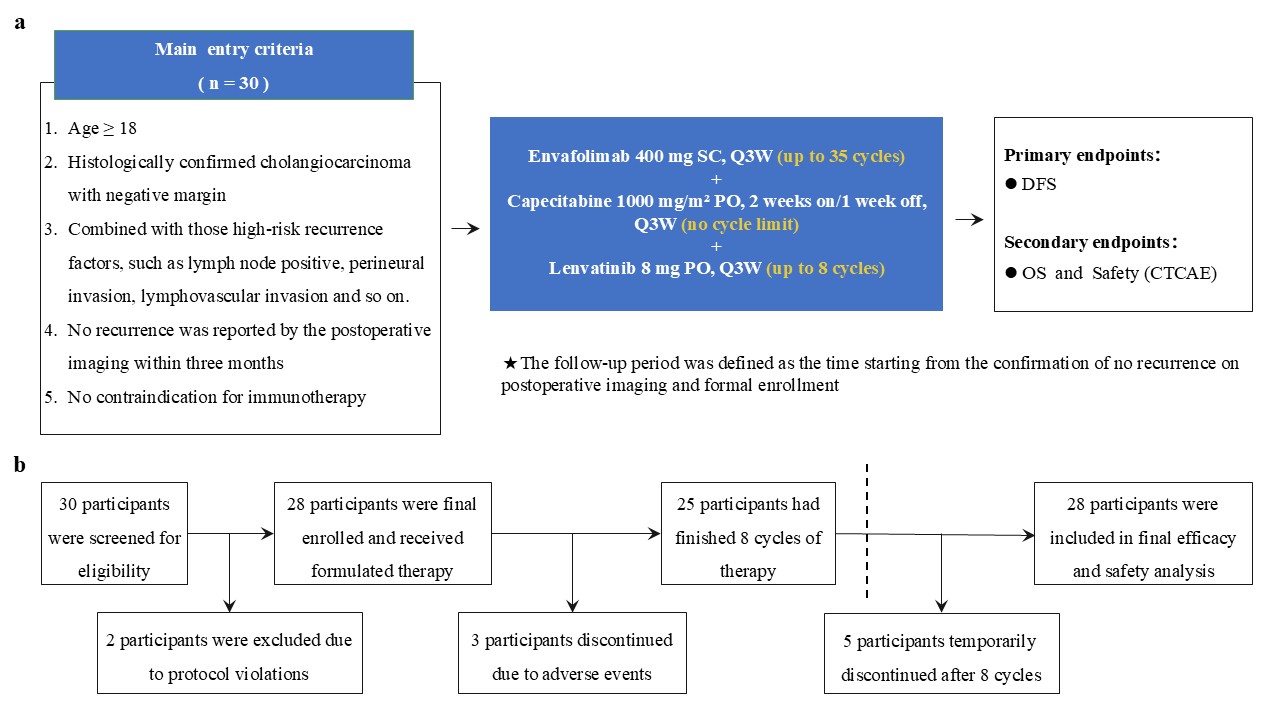


Figure 1. The summary of the study protocol
