# Supplementary material for: Efficacy and Safety of Envafolimab Combined With Capecitabine and Lenvatinib as Postoperative Adjuvant Therapy in Resected Biliary Tract Cancer With High‐Risk Recurrence Factors: A Phase II Single‐Center Prospective Study
Source: Cancer Med. 2026 Mar 27;15(4):e71756. doi: 10.1002/cam4.71756 (PMC13140624; doi:10.1002/cam4.71756)
Supplement: Supplementary file 2 — Table S1: Fisher's exact tests for the relationship between tumor marker status at different time points (before operation/triple therapy initiation/post‐first triple therapy) and ER risk. [file CAM4-15-e71756-s002.docx]

**Supplementary Table 1.** Fisher’s exact tests for the relationship between tumor marker status at different time points (before operation/triple therapy initiation/post-first triple therapy) and ER risk.

| Time | Stratification Variable | Subgroup | Status | Patients (n) | P-value |
| --- | --- | --- | --- | --- | --- |
| Before operation | CA19-9 level | Normal (≤37 U/mL) | ER | 0 | 0.029 |
|  |  |  | Non-ER | 9 |  |
|  |  | Abnormal (>37 U/mL) | ER | 8 |  |
|  |  |  | Non-ER | 11 |  |
| Triple therapy initiation | CA19-9 level | Normal (≤37 U/mL) | ER | 6 | 1.000 |
|  |  |  | Non-ER | 12 |  |
|  |  | Abnormal (>37 U/mL) | ER | 1 |  |
|  |  |  | Non-ER | 4 |  |
| After first triple therapy | CA19-9 level | Normal (≤37 U/mL) | ER | 2 | 0.006 |
|  |  |  | Non-ER | 17 |  |
|  |  | Abnormal (>37 U/mL) | ER | 5 |  |
|  |  |  | Non-ER | 2 |  |
| Before operation | CA125 level | Normal (≤35 U/mL) | ER | 7 | 1.000 |
|  |  |  | Non-ER | 15 |  |
|  |  | Abnormal (>35 U/mL) | ER | 1 |  |
|  |  |  | Non-ER | 4 |  |
| Triple therapy initiation | CA125 level | Normal (≤35 U/mL) | ER | 4 | 0.646 |
|  |  |  | Non-ER | 8 |  |
|  |  | Abnormal (>35 U/mL) | ER | 2 |  |
|  |  |  | Non-ER | 8 |  |
| After first triple therapy | CA125 level | Normal (≤35 U/mL) | ER | 5 | 0.588 |
|  |  |  | Non-ER | 16 |  |
|  |  | Abnormal (>35 U/mL) | ER | 2 |  |
|  |  |  | Non-ER | 3 |  |
| Before operation | CEA level | Normal (≤5 ng/mL) | ER | 6 | 1.000 |
|  |  |  | Non-ER | 13 |  |
|  |  | Abnormal (>5 ng/mL) | ER | 2 |  |
|  |  |  | Non-ER | 6 |  |
| Triple therapy initiation | CEA level | Normal (≤5 ng/mL) | ER | 6 | 1.000 |
|  |  |  | Non-ER | 14 |  |
|  |  | Abnormal (>5 ng/mL) | ER | 1 |  |
|  |  |  | Non-ER | 2 |  |
| After first triple therapy | CEA level | Normal (≤5 ng/mL) | ER | 5 | 0.287 |
|  |  |  | Non-ER | 17 |  |
|  |  | Abnormal (>5 ng/mL) | ER | 2 |  |
|  |  |  | Non-ER | 2 |  |

CA 19-9, carbohydrate antigen 19-9. ER, early recurrence. Non-ER, non-early recurrence. CA125, carbohydrate antigen 125. CEA, carcinoembryonic antigen.
